# Effect of residual myocardial ischemia on recovery of left ventricular function after primary percutaneous coronary intervention

**DOI:** 10.1186/s12872-024-03777-3

**Published:** 2024-03-19

**Authors:** Mohamed Aly Abdelhafez, Karim M. E. Aly, Amr A. A. Youssef

**Affiliations:** 1 Lecturer of Cardiology, Faculty of Medicine, Assiut University, Assiut, 17575 Egypt; 2 Demonstrator of Cardiology, Faculty of Medicine, Assiut University, Assiut, 71515 Egypt; 3 Professor of Cardiology, Faculty of Medicine, Assiut University, Assiut, 17575 Egypt

**Keywords:** 2-D speckle tracking, Primary percutaneous coronary intervention, Left ventricular function, Residual myocardial ischemia, GLS-A2C, GLS-AVG, Single vessel disease, Multiple vessel disease

## Abstract

**Background:**

It is unknown whether the existence of severe bystander damage will affect left ventricular (LV) healing following primary percutaneous coronary intervention (PPCI).

The aim of the present analysis was to follow LV recovery using 2D speckle tracking echocardiography (2-D STE) in cases with single versus multiple vessel disease with acute myocardial infarction (AMI) who underwent PPCI and to assess major adverse cardiovascular events (MACEs) within 3 months.

**Patients and methods:**

This work was conducted at Assiut University Heart Hospital. Of 1026 screened subjects with AMI needing PPCI and assessed for eligibility, only 89 cases fulfilled the inclusion criteria. They were classified into Group A: single vessel and Group B: multiple vessel (≥ 2 vessels) disease. Their data were obtained on admittance and after 90 days.

**Results:**

In group A compared to group B, there was a statistically preferable value at baseline in the global longitudinal strain- Apical 2 chamber (GLS-A2C) (-12.05 ± 3.57 vs. -10.38 ± 3.92, P = 0.039). At follow-up, the improvement was in all 2-D STE variables, including GLS-long axis (GLS-LAX) (-13.09 ± 3.84 vs.-10.75 ± 3.96, P = 0.006), GLS- apical 4 chamber (GLS-A4C) (-13.23 ± 3.51 vs.-10.62 ± 4.08, *P* = 0.002), GLS-A2C (-13.85 ± 3.41 vs-10.93 ± 3.97, *P* < 0.001) and GLS- average (GLS-AVG, P = 0.001). There was a considerable negative correlation between the recovery of LV performance and the existence of multi-vessel lesions (P = 0.009). There was no variance between the groups regarding MACEs.

**Conclusions:**

Patients with single vessel lesions who underwent PPCI to the culprit lesion had better recovery of LV function than those with multi-vessel (≥ 2 vessels) lesions who underwent PPCI to the culprit lesion only. The presence of multivessel involvement was an independent risk factor for deterioration in GLS.

**Trial Registration:**

Registered in clinical trial, clinicalTrial.gov ID NCT04103008 (25/09/2019). IRB registration: 17,100,834 (05/11/2019).

## Introduction

Multi-vascular disease (MVD) is identified in many patients who have AMI. Management of non-culprit lesions in the same setting is debatable; however, it is of greatest importance to treat the culprit lesion at the index surgery. It is unknown whether the existence of severe bystander damage will affect LV healing following PPCI. 2-D speckle tracking echocardiography (2-D STE) has evolved to quantify global longitudinal strain (GLS); it is noninvasive and enables a quantitative, objective assessment of both global and local myocardial performance, independent of the insinuation angle and cardiac translational motions [1, 2].

GLS measured by 2-D STE provides additional prognostic information over echocardiographic left ventricular ejection fraction (LVEF) assessment alone. It is a compelling predictor of LV recovery, residual myocardial ischemia, and cardiac mortality [2–5]. Both measurements are crucial instruments for clinical use since they are easy to use, repeatable and quick.

The current work aimed to use 2-D STE before hospital discharge and after a 3-month follow-up to evaluate LV recovery in patients with single versus multiple artery disease and AMI who underwent PPCI. Furthermore, major adverse cardiovascular events (MACEs) within 90 days (nonfatal MI, stent thrombosis, hospital admission for heart failure, and cardiovascular mortality) were assessed.

## Patients and Methods

### Type of Study, Study duration and setting

This was an observational cohort prospective study that was conducted from August 2021 to August 2022 at Assiut University Heart Hospital.

### Study subject

Of 1026 screened patients with AMI who underwent PPCI and were assessed for eligibility, 89 fulfilled the inclusion criteria and sample size.

### Classification

**Fig. 1:**
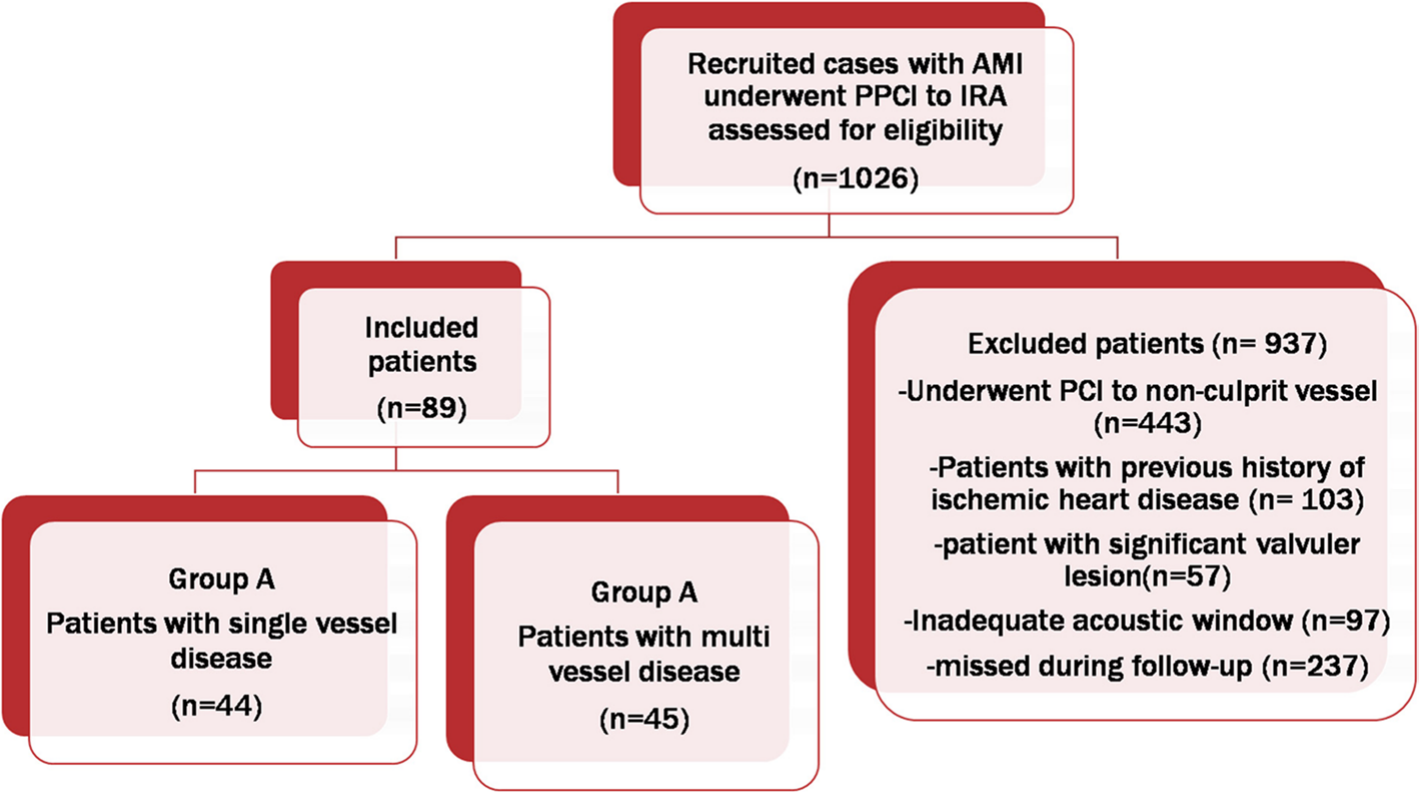
Flowchart of the recruited cases with AMI undergoing PPCI in the study period

The study population was randomly classified into Group A: single vessel disease (*n* = 44) and Group B: multiple vessel (≥ 2 vessels) disease (*n* = 45), as shown in the flowchart in Fig. 1. The data were acquired on admittance and at follow-up after 3 months.

#### a. Inclusion criteria

Cases with AMI eligible for PCI seen on admittance and after 12 weeks, from both genders, aged from 18 to 80 years old.

#### b. Exclusion criteria

Patients with a history of prior ischemic heart disease, cardiomyopathy, considerable valvular lesion (especially mitral regurgitation), cancer, cytostatic therapy, mental disorders, and acute and chronic inflammation. Additionally, patients who were managed by PCI to non-infarct-related arteries before the follow-up period, those with inadequate acoustic windows and unacceptable heart rates during the study and those missed during follow-up were excluded.

#### c. Sample Size Calculation

Using the OpenEpi® sample size calculator at a confidence level of 95% and a 2-D speckle echocardiography sensitivity of 96% and specificity of 78% for the assessment of LV function (3), a total of 76 patients who were eligible for emergency PCI were enrolled. This number was then raised to 89 to account for a 10% drop off.

### Study tools

#### History and examination

Detailed history, demographic, and clinical data were recorded. Full examination, including general examination, height, weight, body mass index, intra-procedural systolic and diastolic blood pressure and heart rate, was performed, as well as cardiac examination to distinguish signs of heart failure.

#### 212 leads ECG

To ensure the diagnosis of acute MI and record the related abnormalities in rate and rhythm.

#### Laboratory investigations

Random blood glucose, serum creatinine, electrolytes and lipid profiles were evaluated before the procedure.

#### Echocardiographic studies

Measurements of LV function by ejection fraction (EF) using 2-Dimentional echocardiography using modified Simpson's method and wall motion score index as well as by 2-Dimentional speckle tracking to assess global longitudinal strain was performed in all patients upon admittance and 3 months post-PCI procedure.

#### Standard echocardiographic technique

Detailed resting echocardiographic assessment was performed on all included subjects according to the latest guidelines [4]. by the *GE Healthcare Ultrasound system and using the Phillips latest ST package (Echo PAC)* for analysis.

#### Speckle Tracking Echocardiography

The following factors were taken into account when taking the picture. For the measurement of LV longitudinal strain, images from the apical four-chamber, two-chamber, and three-chamber perspectives were necessary. The gray scale frame rate was held constant at 30 to 70 frames per second. The appropriate gating of the pictures needed a high-quality ECG signal.

2. Interpretation: The longitudinal strain parameter was more replicable than the radial, circumferential, and rotational strains. Correspondingly, global strain demonstrated substantially better reproducibility than segmental strain. The typical GLS is typically more negative, or between 16 and 18%. Angiographic and procedural methods:

Coronary angiography and PPCI were performed by an interventional cardiologist, a member of the PPCI team, and the following values were retrieved: infarct-related culprit lesions and non-infarct-related culprit lesions and final TIMI flow [5].

### Statistical analyses

Statistical analyses were executed using SPSS version 22.0 (IBM Corporation, Armonk, NY, USA). Variables are presented as the mean ± SD or percentage as indicated. Student’s *t* test or the Mann–Whitney *U* test was used for normally or non-normally distributed values, respectively. The chi-square test was used to evaluate the differences statistically between proportions. *P* < 0.05 was considered statistically significant.

### Ethical Approval

***The trial is registered in clinical trial, clinicalTrial.gov ID*** *NCT04103008* (25/09/2019). Approval of the study from the Faculty of Medicine, Assiut University ethics committee was obtained, IRB number 17100834 **(**05/11/2019**)**. Written informed consent was obtained from the participants or the participants’ caregivers. The privacy of the included subjects was guaranteed, and the confidentiality of all their data was assured.

## Results

A total of 1026 cases were screened during the study period. Patients who had a previous history of ischemic heart disease (*n* = 103), significant valvular lesions (*n* = 57), patients who underwent PCI to non-infarct-related arteries before follow-up (*n* = 443), patients with inadequate acoustic windows and improper heart rates during the study (*n* = 97) and patients missed during follow-up (*n* = 237) were excluded. The included cases with AMI undergoing PPCI were 89 and were classified into 2 groups: Group A (single vessel disease) (*n* = 44) and Group B (multiple vessel (≥ 2 vessels) disease) (*n* = 45). The data were obtained on admittance and at follow-up after 3 months.

The majority (93.2 and 93.3%) of both groups were male, and the mean age was significantly higher in Group B [52.59 ± 9.72 vs. 59.09 ± 11.54, *P* = 0.005]. There was no difference in their smoking habits, marital statuses or occupations. There was no significant difference between the two groups regarding the preliminary clinical examination, including general, chest, and cardiac examinations. Regarding the associated comorbidities in both groups, the most commonly recorded were hypertension (25% and 37.8%, respectively) and diabetes mellitus (40.9% and 31.1%, respectively), with no statistically significant difference in either group. The most common presentation was anterior MI in both groups (77.3% and 73.3%), followed by inferior and lateral MI.

The main echocardiographic findings in recruited patients are summarized in Table 1. There was no considerable variance between the groups at baseline or after 3 months. The follow-up measures recorded significant refinement in EF by M-mode, Simpson's and WMSI in both categories.

**Table 1:** Baseline and 3-month follow-up echocardiographic (ECHO) findings of included patients with AMI undergoing PPCI (*n* = 89)

ECHO	Group A (*n* = 44)	Group B (*n* = 45)	*P* value
**EF M Mode**			
**At baseline**			
Mean ± SD	47.25 ± 7.29	46.18 ± 7.93	0.509
Range	34.0–65.0	36.0–65.0	
**After 3 months**			
Mean ± SD	48.70 ± 7.47	46.98 ± 7.64	0.284
Range	39.0–65.0	38.0–65.0	
***P*** ** value** ^**2**^	< 0.001*	0.002*	
**EF Simpson’s**			
**At baseline**			
Mean ± SD	45.13 ± 7.52	44.61 ± 7.39	0.202
Range	35.0–64.0	34.0–61.0	
**After 3 months**			
Mean ± SD	47.09 ± 7.53	45.60 ± 7.74	0.156
Range	38.0–62.0	33.0–62.0	
***P*** ** value** ^**2**^	< 0.001*	< 0.001*	
**WMSI**			
**At baseline**			
Mean ± SD	1.55 ± 0.27	1.58 ± 0.30	0.544
Range	1.0–2.1	1.1–2.1	
**After 3 months**			
Mean ± SD	1.47 ± 0.26	1.54 ± 0.29	0.194
Range	1.0–1.9	1.0–2.1	
***P*** ** value** ^**2**^	< 0.001*	< 0.001*	

*AMI* Acute myocardial infarction, *PPCI* Primary percutaneous coronary intervention, *EF* Ejection fraction, *WMSI* Wall motion score index

*P* value = difference between single vessel and multiple vessel (≥ 2 vessels) lesions

*P* value^2^ = difference between baseline and after 3 months

*= highly significant

Table 2 discloses the angiographic data of the included participants. There was no recorded significant variance in culprit lesion distribution among both groups and no variance in final TIMI flow between both groups.

**Table 2:** Coronary angiographic changes in included patients with AMI undertaking PPCI (*n* = 89)

	**Group A (** ***n*** ** = 44)**		**Group B (** ***n*** ** = 45)**		***P*** ** value**
	**No**	**%**	**No**	**%**	
**LM**					
Non significance	44	100.0%	45	100.0%	
Significant lesion	0	0.0%	0	0.0%	–
Culprit lesion	0	0.0%	0	0.0%	
**LAD**					
No significance	9	20.5%	4	8.9%	
Significant lesion	0	0.0%	7	15.6%	0.189
Culprit lesion	34	77.3%	34	75.6%	
**Diagonal**					
Non significance	43	97.7%	38	84.4%	
Significant lesion	0	0.0%	7	15.6%	0.016*
Culprit lesion	1	2.3%	0	0.0%	
**LCX**					
Non significance	43	97.7%	33	73.3%	
Significant lesion	0	0.0%	10	22.2%	0.003*
Culprit lesion	1	2.3%	2	4.4%	
**Ramus**					
Non significance	3	100.0%	3	60.0%	
Significant lesion	0	0.0%	2	40.0%	0.464
Culprit lesion	0	0.0%	0	0.0%	
**RCA**					
Non significance	37	84.1%	15	33.3%	
Significant lesion	0	0.0%	22	48.9%	0.193
Culprit lesion	8	18.1%	8	17.8%	
**PDA**					
Non significance	44	100.0%	0	0.0%	
Significant lesion	0	0.0%	45	100.0%	–
Culprit lesion	0	0.0%	0	0.0%	
**OM**					
Non significance	43	97.7%	35	77.8%	
Significant lesion	0	0.0%	9	20.0%	0.016*
Culprit lesion	0	0.0%	1	2.2%	
**Culprit lesion**					
LAD	34	77.3%	34	75.6%	
LAD-Diagonal	0	0.0%	1	2.2%	
RCA	8	18.1%	7	15.6%	0.633
LCX	1	2.3%	2	4.4%	
OM	0	0.0%	1	2.2%	
Diagonal	1	2.3%	0	0.0%	

*2-D* Two-dimensional, *LM* Left main, *RCA* Right coronary artery, *LM* Left main, *LAD *Left anterior descending, *PDA* Posterior descending artery, *LCX* Left circumflex coronary artery, *RASMUS* Ramus intermedius vessel, *OM* Obtuse marginal vessel

*= highly significant

**Fig. 2:**
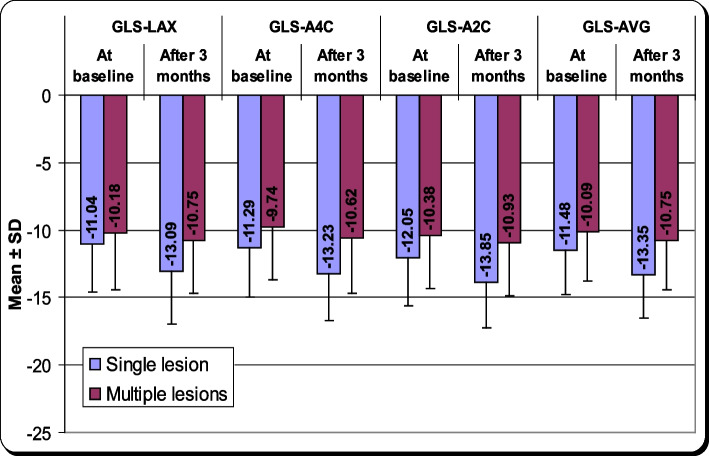
The follow-up 2D-speckel tracking changes in included participants with AMI undergoing primary PCI (*n* = 89)

The baseline and follow-up 2-D speckle tracking variations in included subjects with AMI undergoing PPCI are demonstrated in Table 3 and Fig. 2. In group A compared to group B, there was statistically considerable improvement at baseline in GLS-A2C (-12.05 ± 3.57 vs. -10.38 ± 3.92, *P* = 0.039). After 12 weeks, the improvement was in all 2D-STE variables, including global longitudinal strain-long axis (GLS-LAX) (-13.09 ± 3.84 vs.-10.75 ± 3.96. *P* = 0.006), global longitudinal strain—apical 4 chamber (GLS-A4C) (-13.23 ± 3.51vs. -10.62 ± 4.08, *P* = 0.002), global longitudinal strain apical 2 chamber (GLS-A2C) (-13.85 ± 3.41 vs. -10.93 ± 3.97, *P* < 0.001) and global longitudinal strain-average (GLS-AVG, *P* = 0.001).

**Table 3:** The baseline and follow-up 2D-speckel tracking changes in included participants with AMI undergoing primary PCI (*n* = 89)

	**Group A (** ***n*** ** = 44)**	**Group B (** ***n*** ** = 45)**	***P*** ** value** ^**1**^
	**Mean ± SD**	**Mean ± SD**	
**GLS-LAX:**			
At baseline	-11.04 ± 3.60	-10.18 ± 4.26	0.306
After 3 months	-13.09 ± 3.84	-10.75 ± 3.96	0.006*
*P* value^2^	0.000*	0.091	
**GLS-A4C:**			
At baseline	-11.29 ± 3.70	-9.74 ± 3.95	0.060
After 3 months	-13.23 ± 3.51	-10.62 ± 4.08	0.002*
*P* value^2^	< 0.001*	0.001*	
**GLS-A2C:**			
At baseline	-12.05 ± 3.57	-10.38 ± 3.92	0.039*
After 3 months	-13.85 ± 3.41	-10.93 ± 3.97	< 0.001*
*P* value^2^	< 0.001*	0.083	
**GLS-AVG:**			
At baseline	-11.48 ± 3.26	-10.09 ± 3.69	0.062
After 3 months	-13.35 ± 3.21	-10.75 ± 3.71	0.001*
*P* value^2^	< 0.001*	0.009*	
**HR:**			
At baseline	79.82 ± 14.32	77.20 ± 13.02	0.369
After 3 months	69.95 ± 8.82	71.91 ± 10.25	0.337
*P* value^2^	< 0.001*	0.003*	

*GLS-LAX* Global longitudinal strain- long axis, *GLS-A4C* Global longitudinal strain-Apical 4 chambers, *GLS-A2C* Global longitudinal strain-Apical 2 chambers, *GLS-AVG* Global longitudinal strain- average, *HR* Heart rate

*P* value = Difference between single vessel and multiple lesions

*P* value^2^ = Difference between baseline and after 3 months

*= highly significant

Multiple linear regression analysis displayed a significant negative correlation between the recovery of LV function and the existence of multivessel disease (*P* = 0.009) (Table 4).

**Table 4:** Multiple linear regression analysis for deterioration of the global longitudinal strain-average (GLS AVG)

	**Unstandardized** **coefficients**		**Standardized** **Coefficients**	**T**	***P*** ** value**	**95.0% C.I. for B**	
	**B**	**SE**	**Beta**			**Lower**	**Upper**
**(Constant)**	8.413	12.409		0.678	0.500	-16.272	33.098
**Age (years)**	0.107	0.186	0.064	0.573	0.568	-0.264	0.477
**Gender**	12.258	8.966	0.168	1.367	0.175	-5.578	30.094
**Smoking**	10.818	5.017	-0.262	**-2.156**	0.034*	-20.799	-0.838
**Hypertension**	2.186	4.640	-0.055	-0.471	0.639	-11.415	7.044
**DM**	4.772	4.186	0.125	1.140	0.258	-3.556	13.100
**Multivessel disease**	10.658	3.970	-0.291	**-2.685**	0.009*	-18.556	-2.761

The effect of risk factors on the global longitudinal strain average was demonstrated by multiple linear regression analysis, and there was statistical significance for the existence of multivessel effects (*p* = 0.009). Age, sex, hypertension and diabetes mellitus were not predictors for the worsening of global longitudinal strain **(**Table 5**)**

There was no statistically significant variance among groups regarding MACE (Table 5).

**Table 5:** Frequency of non-fatal adverse events in included participants with AMI treated by primary PCI (*n* = 89)

	**Group A (** ***n*** ** = 44)**		**Group B (** ***n*** ** = 45)**		***P*** ** value**
	**No**	**%**	**No**	**%**	
**Nonfatal MI**	0	0.0%	0	0.0%	–
**Hospital admittance with HF**	5	11.4%	8	17.8%	0.392
**Stent thrombosis**	0	0.0%	0	0.0%	–
**CV death**	0	0.0%	0	0.0%	–

*MI*  Myocardial infarction, *HF*  Heart failure, *CV* Cardiovascular

## Discussion

The main finding of the present study was that in included cases with AMI undergoing PPCI, there was no observed statistically notable variance among both groups (single vs. ≥ 2 vessel lesions) at baseline and after 3 months by using standard trans-thoracic echocardiography. However, the follow-up measures recorded noteworthy refinement in both groups. Using 2D-speckle tracking in group A (single vessel lesion) compared to group B (≥ 2 vessel lesions), there was statistically significant improvement at baseline in GLS-A2C. At follow-up, the improvement was in all speckle tracking parameters, including global longitudinal strain-long axis (GLS-LAX), global longitudinal strain—apical 4 chamber (GLS-A4C), global longitudinal strain apical 2 chamber (GLS-A2C) and global longitudinal strain-average (GLS-AVG). Multiple linear regression analysis displayed a significant negative correlation between the recovery of LV function and multi-vessel disease. The existence of multi-vessel disease was found to be an independent risk factor for the worsening of global longitudinal strain.

According to Liou et al., the results of LVGLS's ability to reveal CAD were satisfactory.in patients with and without CAD, the mean LVGLS values were [-16.5% and -19.7%]. The presence of moderate-to-severe CAD was also detected by aberrant LVGLS with 74.4% sensitivity, 72.1% specificity, 2.9% positive likelihood ratio and 0.35% negative likelihood ratio [6].

Another work examining the ability of LVGLS to ameliorate the development of coronary artery disease in patients with NSTE-ACS and normal global/regional systolic function summarized that GLS was impaired in patients with significant coronary artery stenosis compared to those without (16:73:4% vs. 22:42:9%, *p* 0:001) and that territorial LS could localize coronary stenosis. This implied that GLS had a higher diagnostic value than the visual echocardiographic wall motion indicator. GLS > 19.7% additionally showed 81% sensitivity and 88% specificity for detecting substantial stenosis.

Radwan and Hussein [8]. presented a lower cutoff for GLS (GLS > 15:6% had AUC 0.88, 95% for the prediction of significant CAD; *p* 0:001) and demonstrated a decrease in GLS parallel with an increase in the number of coronary vessels involved in patients with stable angina and a considerable positive correlation between GLS and LV ejection fraction (EF).

In the current work, it was established that comparing the improvement in Group A vs. Group B after 3 months exhibited that Group A had more obvious variations in GLS-LAX, GLS-A4C, GLS-A2C and GLS-AVG. In accordance, Pastore and colleagues anticipated that the evaluation of patients with acute and chronic CAD using STE would ameliorate the predictive judgment of these cases [7].

Moreover, STE exhibited an association with after-ACS event clinical outcome in diverse studies: LVGLS [< − 13%] measured during the index hospitalization was a forecaster of event-free survival in a cohort of AMI patients [9]. while LVGLS [> − 14%] predicted admittances for acute HF and cardiovascular deaths in AMI sufferers [10].

In a large study of patients with AMI, LVGLS was ominously and independently correlated with all-cause mortality, re-infarction, revascularization, and HF hospitalization at the 3-year follow-up [OR 4:5 for LVGLS (< − 15:1%) and 4.4 for LV strain rate (> − 1:06 s -1), and LVGLS was superior to LV EF after multivariate analysis] [11].

There is consensus that early detection of residual ischemic injury and myocardial viability following AMI may aid in optimizing therapeutic management to avoid complications. The 94 patients with AMI and 137 patients with stable CAD, all of whom had undergone coronary revascularization, were included in a prospective study that supported these findings. The results showed that in stable CAD patients, the addition of endocardial LVGCS (> 20%) to baseline features and EF into a regression model significantly improved the prediction of cardiac events [sensitivity (79%), specificity (84%)] [12].

Moreover, a large pool of data from 9 RCTs involving 2,176 patients was published. In mixed-comparison models, CR-IP (complete revascularization-index procedure) decreased the risk of MACE, recurrent MI, revascularization, and CV mortality with IRA-OR (infarct-related artery-only revascularization). Similarly, in a direct comparative meta-analysis, CR-IP had a 66% lower risk of MI than IRA-OR. Between the two revascularization techniques, there were no differences in all-cause mortality [13]. This meta-analysis showed that CR, either during PPCI or as a staged technique, resulted in lower incidences of MACE, revascularization, and CV mortality than IRA-OR in patients with AMI and multi-vessel disease [13].

Angiographically, there was no recorded significant contrast in culprit lesion distribution in either group. After PCI, comparing the refinement after revascularization in both groups after 3 months revealed that single vessel disease cases (Group A) had more noticeable changes in GLS-LAX, GLS-A4C, GLS-A2C and GLS-AVG. Single vs. multi-vessel PCI in AMI patients has always been debatable, with some reports claiming a preferable clinical outcome from complete revascularization during PPCI [14], while other studies found that the potential harm outweighed the benefits for PCI to non-IRA during primary PCI [15–17].

Complete revascularization should be taken into consideration, with non-IRA addressed either at the index procedure or another time before hospital discharge, according to the ESC 2017 recommendations [18]. On the other hand, the 2012 report said that only IRA should be treated and did not propose total revascularization [19].

Numerous studies have supported these assertions. Retrospective analysis was performed on a cohort of consecutive patients with AMI and MVD treated with PPCI between 2004 and 2008 in 6 tertiary care facilities. The frequency of peri-procedural MI/reinfarction was considerably superior in the CR group compared to the IncR group in terms of mortality and repeat revascularization throughout the hospital stay (13.9% vs. 3.1%, respectively). In the CR group, the incidence of MACEs was considerably greater (14.1% vs. 9.1%, *P* = 0.017). This study's key findings were that early-stage angiographically guided PCI of non-IRAs followed by PPCI on IRA was linked to an increased incidence of in-hospital MACE, primarily peri-procedural MIs/re-infarctions [20].

Another research group conducted a meta-analysis to compare the outcomes of MV-PCI with IRA-PCI during the index PPCI in AMI patients. A total of 12.2% of patients received MV-PCI, while 85.8% underwent IRA-PCI. All-cause mortality, the main endpoint, was substantially higher in the MV-PCI group (8.5% vs. 5.4% in the IRA-PCI group). The MV-PCI group also had lower rates of re-infarction and revascularization. Only 14.2% of patients received MV-PCI during the index procedure, making it challenging to draw firm conclusions from the analysis [21].

In patients with acute MI and multi-vessel disease, fractional flow reserve (FFR)-based selection is preferred to routine angiography-based selection, according to the latest guidelines published in 2022 [22]. However, the FLOWER-MI trial compared complete revascularization by means of FFR- or angiography-guided design and stated that FFR-guided PCI was not linked to a decreased risk of mortality, MI or urgent revascularization at 12 months and carried an insignificant higher probability of MI in the FFR group [23]. The most recent ESC recommendations 2023 affirmed that in hemodynamic stable patients presenting with STEMI and MVD, complete revascularization is the recommended procedure either during the index PCI or within 45 days [24–27]. and the selection of the non-IRA is mainly based on angiographic severity [23, 27].

Similar to the present study, authors demonstrated that the Apical 2 and 4 global longitudinal strain from STE were able to detect early impaired cardiac function, compared with LV ejection fraction from conventional echocardiography in septic shock and sepsis [28, 29].

Lastly, multiple linear regression analysis displayed a non-significant correlation between the recovery of LV function and gender. In contrast, gender differences in the pathophysiology, cardiovascular risk factors, and diagnosis of coronary artery disease are well established. Experimental reports claimed that plaque composition and burden differ by gender and female gender is associated with worse outcomes in the case of ischemic heart disease and, compared with men. They added that women were less likely to undergo interventional cardiac procedures and sustain worse outcomes [30].

### Limitations

Some limitations of the current study also need to be acknowledged.

1. It was a single-center analysis.
2. The follow-up period was short.
3. Difficulty in performing STE in some participants due to inadequate echo window.
4. The sample size was relatively small due to the non-adherence of some cases to the follow-up.
5. Due to the COVID-19 pandemic, restrictions were placed on all unnecessary, nonemergency procedures all over the world except under strict measures and after published guidelines to address each disease.
6. Many recruited patients underwent PCI to non-IRA before 3 months, rendering them ineligible for the study.
7. We did not exclude complicated procedure, which may affect outcome even with complete revascularization.

## Conclusions

Patients with single vessel disease who underwent PPCI to culprit lesion had better recovery in left ventricular function compared with ≥ 2 vessels, who underwent PPCI to culprit lesion only and with remaining residual myocardial ischemia. The existence of multivessel illness was found to be a risk factor for the deterioration of the global longitudinal strain average.

## Data Availability

The datasets used and/or analyzed during the current study are available from the corresponding author upon reasonable request.

## Abbreviations

2 D: Two dimensional
ACS: Acute coronary syndrome
AMI: Acute myocardial infarction
CAD: Coronary artery disease
CR: Complete revascularization
CV: Cardiovascular
CX: Circumflex coronary artery
ECG: Electrocardiogram
EF: Ejection fraction
ESC: European society of cardiology
FFR: Functional flow reserve
GLS: Global longitudinal strain
GLS-A2C: Global longitudinal strain-Apical 2 chambers
GLS-A4C: Global longitudinal strain-Apical 4 chambers
GLS-AVG: Global longitudinal strain- average
GLS-LAX: Global longitudinal strain- long axis
HF: Heart failure
HR: Heart rate
ICA: Internal carotid artery
LA: Left atrium
LAD: Left anterior descending coronary artery
LCX: Left circumflex coronary artery
LM: Left main coronary artery
LS: Longitudinal strain
LV: Left ventricle
MACE: Major adverse cardiac events
MI: Myocardial infarction
MVD: Multi vessel disease
Non-IRA: Non infarct related artery
NSTE-ACE: Non-ST segment elevation-acute coronary syndrome
OM: Obtuse marginal coronary artery
PDA: Posterior descending artery
PPCI: Primary Percutaneous coronary intervention
RAMUS: Ramus intermedius vessel
RCA: Right coronary artery
RCT: Randomized controlled trial
RF: Radio frequency
RV: Right ventricle
SPECT: Single photon emission computed tomography
SR: Strain rate
ST segment: ECG section between the end of S wave and beginning of T wave
STE: Speckle tracking echocardiography
STE-ACS: ST-elevation ACS
STEMI: ST segment elevation myocardial infarction
STI: Speckle tacking imaging
WMSI: Wall motion score index
